# The mobile phone addiction index: Cross gender measurement invariance in adolescents

**DOI:** 10.3389/fpsyg.2022.894121

**Published:** 2022-07-18

**Authors:** Xianli An, Siguang Chen, Liping Zhu, Caimin Jiang

**Affiliations:** ^1^Department of Psychology, School of Educational Science, Yangzhou University, Yangzhou, China; ^2^Psychological Education Center, Guangzheng Preparatory School, Dongguan, China

**Keywords:** Mobile Phone Addiction Index, measurement invariance, middle school students, adolescents, gender

## Abstract

The Mobile Phone Addiction Index (MPAI) is a short instrument to assess mobile phone addiction. The Chinese version of this scale has been widely used in Chinese students and shows promising psychometric characteristics. The present study tested the construct validity and measurement invariance of the MPAI by gender in middle school adolescents. The data were collected from 1,395 high school students (females, *n* = 646; *M* age = 15.3 years). Confirmatory factor analysis (CFA) and multiple-group CFA (MG-CFA) for invariance tests were conducted on the MPAI model which consisted of 17 observed items and 4 latent factors. Findings showed that the data fit the four-factor structure model well for both males and females. Furthermore, configural, metric, scalar, and residual invariance were established by gender. The results indicated that the MPAI has acceptable psychometric properties when used in adolescents. In addition, with the strict invariance requirements being satisfied, the underlying factor scores for MPAI can be meaningfully compared across genders. To our knowledge, this study is the first attempt to test the measurement invariance of the MPAI across male and female adolescents. Our results will support future research on mobile phone addiction in adolescents.

## Introduction

According to a survey by the China Internet Network Information Center, 99.7% of Chinese internet users were using smartphones to get online until December 2021 when the survey took place. Meanwhile, the proportion using computers, tablets, and TVs decreased (China Internet Network Information Center [CNNIC], 2021). This suggests that the problem of mobile phone addiction and mobile phone dependence is becoming more serious. Although there are debates about the concepts, criteria, and methodologies used in relation to understanding mobile phone addiction, the fact remains that mobile phones allow problematic and/or compulsory use (José et al., 2016). Given that the abuse of mobile phones can lead to physical, psychological, and behavioral problems, and the behavioral characteristics of problematic mobile phone use have many similarities with gambling disorder and substance addiction, some researchers consider mobile phone addiction to be one of the greatest addictions of the current century (Shambare, 2012), and excessive attention and uncontrolled dedication to one’s mobile phone should be considered mobile phone addiction (José et al., 2016).

The 27-item Mobile Phone Problem Use Scale (MPPUS) was the earliest developed and one of the most widely used instruments for measuring mobile phone overuse or mobile phone addiction/dependency (Bianchi and Phillips, 2005). The MPPUS addresses five addictive symptoms and shows excellent internal consistency and validity in adults. However, it consists of many items that may be somewhat redundant, and this may be a problem when conducting research on adolescents (Foerster et al., 2015). Leung (2008) modified the MPPUS to develop the Mobile Phone Addiction Index (MPAI), which consists of only 17 items. The MPAI has been shown to be a suitable instrument for measuring problematic mobile phone use in adolescents (Leung, 2008), with a high degree of reliability and excellent validity (Liu et al., 2017; Li et al., 2021; Zhao et al., 2021).

In 2014, Huang et al. (2014) adapted the MPAI for use in Chinese participants. The psychometric analyses showed good construct validity and high reliability when used with Chinese undergraduate students. Nonetheless, the use of the MPAI in Chinese middle school adolescents is not as widespread as in college students. In contrast, the internet penetration rate of middle school students in China is close to that of adults, reaching more than 98%. Moreover, about 92% of middle school students in China use mobile phones as their primary means for accessing the internet (HLWXZBG, 2021). In addition, adolescents are in a critical developmental period in which they are more susceptible to addictive behaviors (Squeglia and Gray, 2016). Compared to adults, adolescents spend longer durations engaged in smartphone use and there is a higher prevalence of smartphone addiction (Hwang and Park, 2017). This can lead to negative consequences in relation to the emotional status, academic performance, and interpersonal relationships of teenagers (Lemola et al., 2015; Samaha and Hawi, 2016). Therefore, it is necessary to properly measure mobile phone addiction in adolescents.

In mobile phone addiction research, comparison according to gender is a common concern. For example, when spending time on mobile phones, it is known that males prefer playing mobile games while females prefer watching online videos, listening to music, online shopping, and using social media (Bianchi and Phillips, 2005; Choi et al., 2015). There are also differences between males and females in the prevalence of mobile phone addiction (José et al., 2016). Therefore, an issue arises: are the MPAI scores comparable between male and female adolescents?

Comparisons of observed scores between males and females should be based on the measurement invariance at the latent level across gender (Cai et al., 2008). However, most previous studies assessing the internal consistency, structural validity, criterion validity, and predictive validity of the MPAI have relied solely on classical test theory. Furthermore, studies that were split by gender also lacked confirmatory factor analyses. The comparison of observed scores between males and females is inappropriate if the scale only meets the classical test theory demand. To our knowledge, there is only one study demonstrating that the observed scores of the MPAI met the requirement of measurement invariance across gender (Chen et al., 2021). However, only college students were recruited in this study and as such, the cross-gender measurement invariance of the MPAI is not yet established in adolescents. Therefore, in the present study, we aimed to validate the four-factor structure and to determine the measurement invariance across gender of the MPAI when used in adolescents. It was intended that this would help provide a practical tool for adolescents that can be reliably deployed in measuring mobile phone addiction.

## Materials and methods

### Participants

We employed a cross-sectional study design and a cluster convenience sampling method. The study was conducted between September and December 2021 and data collection took place at schools during mental health education classes and class meeting hours. Participants were provided with information about the study and were informed that they could refuse to participate or withdraw from the study at any time. All students verbally provided informed consent, parents provided passive informed consent as per Maenhout et al. (2020). They were assured that their responses would be confidential and that no information would be shared further. Participants did not receive any reward for participation. One hundred and thirteen students declined to participate. On completion, 1,395 complete questionnaires were obtained (females, *n* = 646). Participants were aged between 12 and 19 years (*M* = 15.30; *SD* = 1.67) recruited across six grades of lower-secondary (50.3%) and higher-secondary (49.7%) schools in Anhui and Guangdong province, China. Ethics approval for this study was obtained from the Research Ethics Committee of the School of Educational Science, Yangzhou University (JKY-2021030401).

### Measure

The Mobile Phone Addiction Index (MPAI) (Huang et al., 2014) was employed to measure mobile phone addiction. This scale contains 17 items and 4 subscales: Inability to Control Craving, Anxiety and Feeling Lost, Withdrawal and Escape, and Productivity Loss. Each item of the MPAI was rated on a 5-point Likert scale (with 1 = not at all and 5 = always). A higher MPAI score reflects greater levels of mobile phone addiction. The Chinese version of the MPAI has satisfactory reliability and validity when used in students (Huang et al., 2014; Lian et al., 2016; Han et al., 2017; Hao et al., 2019).

### Data analyses

Statistical analyses involved: (a) Calculation of descriptive statistics and the Mann–Whitney *U*-test to examine and compare the distributions of the survey responses of males and females; (b) CFA to assess the model fit of the four-factor structure of MPAI in the total sample and each independent group; (c) Ordered categorical MG-CFA to test the gender invariance; and (d) Cross-gender comparisons of latent means. SPSS 28.0 was used to perform the descriptive analyses, Mann–Whitney *U*-test, and internal consistency of Cronbach’s alpha coefficients. CFA and MG-CFA analyses were performed with Mplus 8.7. The weighted least squares mean and variance (WLSMV) adjusted estimator with theta parameterization was used to provide the best solution for modeling ordinal Likert-type data models (Beauducel and Herzberg, 2006).

First, the descriptive analysis of the item response distributions was conducted. Response percentages were calculated for each categorical variable. Then the response differences to each item across gender were compared using the non-parametric Mann–Whitney *U*-test with α set to 0.05.

For the CFA, given the limitations that χ^2^ (or Δχ^2^) is sensitive to rejection of the null hypothesis with large sample sizes and complex models (Cheung and Rensvold, 2002), a liberal χ^2^/*df* ratio was used as a model fit index. The following fit indices were taken to indicate acceptable fit for the CFA: χ^2^/*df* < *5*, CFI (comparative fit index) > 0.90, TLI (Tucker–Lewis fit index) > 0.90, RMSEA (root mean square error of approximation) < 0.08, and SRMR (standardized root mean square residual) < 0.06 (Hu and Bentler, 1999; Hooper et al., 2008). The reliability of the MPAI was evaluated by using internal consistency measures of Cronbach’s alpha and McDonald’s Omega.

Measurement invariance testing was then assessed through increasingly restrictive MG-CFA across gender. The testing process was performed according to the Mplus user’s guide (Muthén and Muthén, 2017). The invariance testing process began with the well-fitting baseline model (configural invariance). In the baseline model, the same factorial structure was specified for each group, and no equality constraints were imposed on model parameters across gender. If configural invariance held, the resulting factor solution was evaluated further. Measurement invariance of categorical modeling does not require the latent factor variance, covariance, or means to be invariant. Many researchers propose that strong invariance is sufficient to ensure valid comparisons between groups (Desa, 2018). However, Meredith (1993) argues that strict invariance is required for fair and equitable comparisons. Therefore, configural invariance, metric invariance (weak invariance), scalar invariance (strong invariance), and residual invariance (strict invariance) were estimated. Statistically, the restriction from the four nested models was enhanced step by step. A significant χ2 difference test (DIFFTEST) indicated a significantly different model fit of the nested models. And, if ΔTLI > –0.01, ΔCFI > –0.01, ΔRMSEA < 0.01, and ΔSRMR < 0.010, measurement equivalence of the models should be accepted (Chen, 2007; Desa, 2018).

When residual invariance was established, latent means were compared across gender. When the latent mean differences were examined, the group of males served as the reference group with means of the latent factor fixed to zero. Each latent mean for females represented the difference between the two groups. Thus, a significant mean for the female group indicated a different level of latent factor relative to the male group.

## Results

### Item survey response distributions

**FIGURE 1:**
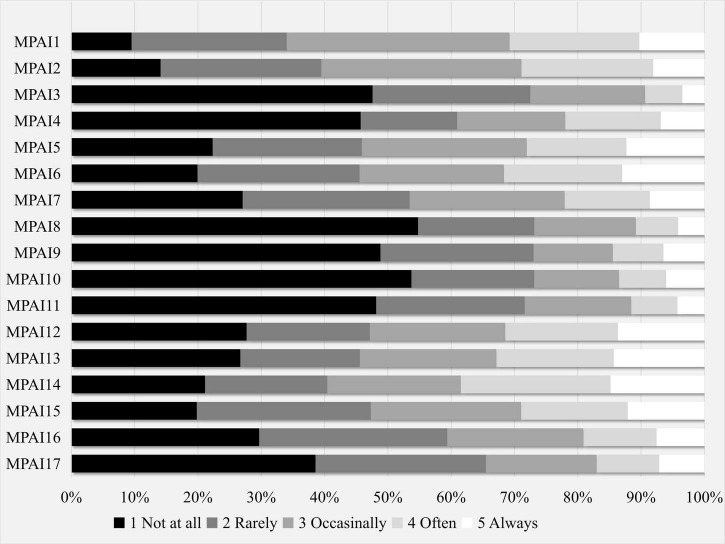
Distributions of item survey responses.

There were five response options for each item and these response option distributions were examined for each item individually (Figure 1). Mann–Whitney *U*-tests found significantly different response distributions for MPAI2 (*Z* = –2.680, *p* = 0.007), MPAI3 (*Z* = –3.872, *p* < 0.001), MPAI4 (*Z* = –3.230, *p* = 0.001), MPAI10 (*Z* = –3.369, *p* < 0.001), MPAI14 (*Z* = –2.149, *p* = 0.032), MPAI15 (*Z* = –3.339, *p* < 0.001), MPAI16 (*Z* = –2.592, *p* = 0.010), and MPAI17 (*Z* = –2.735, *p* = 0.006). Males had higher mean ranks of response than females on these items.

### Confirmatory factor analysis

The CFA was conducted on the total sample to confirm the factor structure of the MPAI. The fit indices (Table 1) were: χ^2^/*df* = 12.742; TLI = 0.907; CFI = 0.923; SRMR = 0.052; RMSEA = 0.092 with an interval at 90% (0.088-0.096). Results showed acceptable TLI, CFI, and SRMR. However, the χ^2^/*df* ratio and RMSEA were poorly fitted and the goodness fit indices showed unsatisfactory model fit. The modification index of the CFA suggested that the error variance between item 1 (MPAI1: “You have been told that you spend too much time on your mobile phone”) and item 2 (MPAI2: “Your friends and family complained about your use of the mobile phone”) should be correlated. The high error correlation between MPAI1 and MPAI2 was consistent with the findings of Chen et al. (2021). Similar wording, content, or directionality may be the reasons for the measurement error covariance between the two items (Zhou et al., 2019).

**TABLE 1:** Goodness of fit indices for total sample and subgroups.

	χ^2^	χ^2^/*df*	TLI	CFI	RMSEA	SRMR
**Male (*n* = 749)**						
Model without error correlation	795.12***	7.036	0.908	0.924	0.090	0.053
Model with error correlation	537.45***	4.799	0.942	0.952	0.071	0.047
**Female (*n* = 646)**						
Model without error correlation	732.40***	6.481	0.910	0.925	0.092	0.057
Model with error correlation	559.47***	5.000	0.935	0.946	0.079	0.053
**Total sample (*n* = 1395)**						
Model without error correlation	1439.92***	12.742	0.907	0.923	0.092	0.052
Model with error correlation	986.16***	8.805	0.938	0.949	0.075	0.046

***p < 0.001.

After inclusion of the error correlation between MPAI1 and MPAI2, except for the inadequate fit of χ^2^/*df* = 8.805, the model fit was improved considerably and was then acceptable: TLI = 0.938; CFI = 0.949; SRMR = 0.046; RMSEA = 0.075 with an interval at 90% (0.071–0.079). Factor loadings of 16 out of 17 items (λ = 0.393 for MPAI 4) were above 0.5 related to the latent factor and statistically significant (*p* < 0.001, Table 2). Supplementary Table 1 shows the polychoric correlations of the items. The fitting of the model also reached the proposed standard in both males (χ^2^/*df* = 4.799, TLI = 0.942; CFI = 0.952; RMSEA = 0.071; SRMR = 0.047) and females (χ^2^/*df* = 5.000, TLI = 0.935; CFI = 0.946; RMSEA = 0.079; SRMR = 0.053) after the same error terms were modified. These findings suggest that the four-factor model of the MPAI was acceptable. In addition, the four subscales presented acceptable of good reliability: The coefficients for Cronbach’s alpha and McDonald’s Omega were all above 0.7 (Table 2).

**TABLE 2:** Factor loadings obtained in the CFA and internal consistency of MPAI.

	Cronbach’s α	McDonald’s ω	Unstandardized		Standardized		*p*
			Estimate	S.E.	Estimate	S.E.	
Inability to control craving	0.782	0.767					
MPAI 1			1.000		0.534	0.023	0.000
MPAI 2			1.110	0.044	0.592	0.021	0.000
MPAI 3			1.120	0.062	0.598	0.023	0.000
MPAI 4			0.736	0.058	0.393	0.026	0.000
MPAI 5			1.302	0.062	0.695	0.018	0.000
MPAI 6			1.408	0.066	0.751	0.016	0.000
MPAI 7			1.297	0.063	0.692	0.018	0.000
Anxiety and feeling lost	0.746	0.826					
MPAI 8			1.000		0.562	0.024	0.000
MPAI 9			1.263	0.061	0.711	0.020	0.000
MPAI 10			1.408	0.067	0.792	0.017	0.000
MPAI 11			1.480	0.068	0.833	0.015	0.000
Withdrawal and escape	0.793	0.823					
MPAI 12			1.000		0.676	0.019	0.000
MPAI 13			1.344	0.042	0.909	0.012	0.000
MPAI 14			1.243	0.038	0.841	0.014	0.000
Productivity loss	0.721	0.801					
MPAI 15			1.000	0.000	0.718	0.019	0.000
MPAI 16			1.075	0.037	0.772	0.017	0.000
MPAI 17			0.981	0.038	0.704	0.019	0.000

### Measurement invariance testing

The configural invariance model was tested first as baseline model. As shown in Table 3, all the fit indices met the satisfactory levels: χ^2^/*df* = 4.900; TLI = 0.939; CFI = 0.949; SRMR = 0.050; RMSEA = 0.075 with an interval at 90% (0.070–0.079). These results indicated that the configural invariance was supported.

**TABLE 3:** Fit indices for measurement invariance models across gender.

	χ^2^	χ^2^/*df*	TLI	CFI	RMSEA	SRMR
Configural	1097.15***	4.900	0.939	0.949	0.075	0.050
Metric	1120.18***	4.726	0.941	0.949	0.073	0.050
Scalar	1118.90***	3.940	0.954	0.952	0.065	0.051
Residual	1108.00***	3.681	0.958	0.953	0.062	0.052
	**χ^2^ Difference test**	***df***	**ΔTLI**	**ΔCFI**	**ΔRMSEA**	**ΔSRMR**
Metric vs. configural	36.248***	13	0.002	0.000	–0.002	0.000
Scalar vs. metric	97.541***	47	0.013	0.003	–0.008	0.001
Residual vs. scalar	51.677***	17	0.004	0.001	–0.003	0.001

***p < 0.001.

The configural model was then compared against the more restrictive metric invariance model. In the metric invariance model, the item loadings were constrained as invariant between males and females. The results showed that the changes resulting from fitting indices between the metric and configural invariance models were not significant (ΔTLI = 0.002 > –0.01; ΔCFI = 0.000 > –0.01; ΔRMSEA = –0.002 < 0.01; ΔSRMR = 0.000 < 0.01), suggesting the weak invariance was confirmed.

Based on the results of configural and metric invariance models, the scalar invariance model was tested to detect whether the thresholds of latent response were equal between males and females. The thresholds and factor loadings were constrained to be equal across gender. The changes resulting from fitting indices between scalar and metric invariance models were not significant (ΔTLI = 0.013 > –0.01; ΔCFI = 0.003 > –0.01; ΔRMSEA = –0.008 < 0.01; ΔSRMR = 0.001 < 0.01), indicating strong invariance between males and females was satisfied.

Subsequently, the residual invariance model was established further. In addition to factor loadings and thresholds were constrained to be equal across gender, the residual variances for the categorical observed items in the two groups were both fixed at unity. The results of ΔTLI = 0.004 > –0.01; CFI = 0.001 > –0.01; ΔRMSEA = –0.003 < 0.01; Δ SRMR = 0.001 < 0.01 indicated that strict invariance was supported.

To further compare the estimated categorical MG-CFA parameters by gender, the results of the factor loadings are presented in Supplementary Table 2.

### Comparison of factor means for males and females

Since the factor loadings, thresholds, and residual variances of the items were invariant across genders, the potential latent mean differences between groups were examined. Table 4 presents the unstandardized latent mean differences, with males as the reference group. The negative values suggested that the female group had lower scores. The latent means for the subscales of Anxiety and Feeling Lost (Estimate = –0.134, *p* = 0.004), and Withdrawal and Escape (Estimate = –0.237, *p* = 0.000) in the female group were significantly lower than in the male group. These results suggest that males may have higher levels of mobile phone addiction than females.

**TABLE 4:** Gender differences in unstandardized latent means of MPAI.

		Estimate	S.E.	Est./S.E.	*p*
Inability to control craving	Male	0.000	0.000		
	Female	–0.049	0.039	–1.259	0.208
Anxiety and feeling lost	Male	0.000	0.000		
	Female	–0.134	0.046	–2.920	0.004
Withdrawal and escape	Male	0.000	0.00		
	Female	0.060	0.055	1.103	0.270
Productivity loss	Male	0.000	0.000		
	Female	–0.237	0.066	–3.616	0.000

## Discussion

In this study, the reliability and construct validity of MPAI was first tested in adolescents. The results showed that the scale has acceptable reliability and good structural validity, consistent with the existing research (Li et al., 2021), and in agreement with previous reports that the application of MPAI in middle school adolescents is appropriate (Liu et al., 2017; Zhao et al., 2021).

We then examined the measurement invariance of the MPAI across gender. The results showed that the configural invariance was met, suggesting that both the males and females have the same four-factor structure of the MPAI. Meanwhile, complete factor loading invariance was satisfied between males and females, indicating that the MPAI items are equally predicted by latent factors across gender. Further, the threshold invariance was also confirmed, implying there is no latent response bias to items on the MPAI between males and females (Reise et al., 1993). The threshold invariance findings also imply that the mean difference of each factor may be directly compared between males and females (Vandenberg and Lance, 2000). Moreover, the residual invariance across gender was confirmed, suggesting the explained variances of extraneous variables for each item are homogeneous across gender. Our results are consistent with the findings obtained by Chen et al. (2021), although they tested the measurement invariance of the MPAI in college students. These findings converge to suggest that the observed MPAI item and factor scores can be directly compared between males and females when used in adolescents.

Based on the results of the measurement invariance testing, we compared the differences in the latent factor means of the MPAI. It was found that males have higher scores for the subscales of Anxiety/Feeling Lost and Productivity Loss than females. However, no significant difference was found for Inability to Control Craving and Withdrawal/Escape scores. The comparisons of the item survey responses across gender also partially support these observed differences. Males had higher mean response ranks on many items than females. For the MPAI, the higher response scores indicate a higher tendency toward addiction. Therefore, the item response distribution also indicates that male adolescents exhibited a higher level of mobile phone addiction than females. These results are partly consistent with some previous studies. For example, while several studies have shown that males and females in middle school have the same overall levels of mobile phone addiction (Buctot et al., 2020; Sun et al., 2020; Zhao et al., 2021) other researchers found that the degree of mobile phone addiction in males is higher than in females (Li and Hao, 2019), and males are more prone to have mobile phone addiction problems than females (Bai et al., 2020). Reasons for the discrepancy between these studies may be due to different study participants and measurement instruments. For instance, some studies included both middle school students and primary school students (Bai et al., 2020; Sun et al., 2020; Zhao et al., 2021), whereas others only used middle school students (Li and Hao, 2019; Buctot et al., 2020). Several studies have used the MPAI as a measurement tool (Li and Hao, 2019; Bai et al., 2020; Sun et al., 2020; Zhao et al., 2021), but others have used different mobile phone addiction or mobile phone dependency-related scales (Buctot et al., 2020).

There are some inevitable limitations in this study. First, the convenience sample of adolescents attending middle schools in this study may limit the generalization of the results. The data were obtained only from two provinces in China and should be repeated with a larger and more diverse population of adolescents from other geographical areas. Second, this study used the self-report scale to assess mobile phone addiction, which may lead to reporting biases. Finally, we did not use other instruments to verify the validity of using the MPAI in adolescents.

## Conclusion

In conclusion, this study confirmed the four-factor structure of the MPAI is retained when used in middle school adolescents. Moreover, the strict measurement invariance of the MPAI was satisfied across gender, indicating that the MPAI factor scores can be obtained by measuring scale items. As such, scores between adolescent males and females can justifiably be compared.

## Data Availability Statement

The original contributions presented in this study are included in the article/Supplementary Material, further inquiries can be directed to the corresponding author/s.

## Ethics statement

The studies involving human participants were reviewed and approved by the Research Ethics Committee of School of Educational Science, Yangzhou University. Written informed consent for participation was not provided by the participants’ legal guardians/next of kin because: (1) The items of the scale used in this study has been viewed and permitted by the schools. (2) The students have been informed that they can decline the investigating process at any time if they want. (3) The MPAI scale used in this study is short and no harm reported when used in Chinese adolescents.

## Author contributions

XA and SC contributed to the conceptualization design for the study and wrote the manuscript. LZ and CJ collected the data. CJ analyzed the data under the guidance of XA. All authors approved the final version of the manuscript for submission.

## Conflict of Interest

The authors declare that the research was conducted in the absence of any commercial or financial relationships that could be construed as a potential conflict of interest.

## Publisher’s Note

All claims expressed in this article are solely those of the authors and do not necessarily represent those of their affiliated organizations, or those of the publisher, the editors and the reviewers. Any product that may be evaluated in this article, or claim that may be made by its manufacturer, is not guaranteed or endorsed by the publisher.
