# Supplementary material for: The mobile phone addiction index: Cross gender measurement invariance in adolescents
Source: Front Psychol. 2022 Jul 18;13:894121. doi: 10.3389/fpsyg.2022.894121 (PMC9340052; doi:10.3389/fpsyg.2022.894121)
Supplement: Supplementary file 1 [file Data_Sheet_1.docx]

Supplementary Material

# Supplementary Tables

**Table 1. Polychoric correlation matrix of MPAI items for total sample.**

|  | 1 | 2 | 3 | 4 | 5 | 6 | 7 | 8 | 9 | 10 | 11 | 12 | 13 | 14 | 15 | 16 |
| --- | --- | --- | --- | --- | --- | --- | --- | --- | --- | --- | --- | --- | --- | --- | --- | --- |
| 1.MPAI1 |  |  |  |  |  |  |  |  |  |  |  |  |  |  |  |  |
| 2.MPAI2 | 0.659 |  |  |  |  |  |  |  |  |  |  |  |  |  |  |  |
| 3.MPAI3 | 0.362 | 0.400 |  |  |  |  |  |  |  |  |  |  |  |  |  |  |
| 4.MPAI4 | 0.239 | 0.263 | 0.305 |  |  |  |  |  |  |  |  |  |  |  |  |  |
| 5.MPAI5 | 0.355 | 0.391 | 0.413 | 0.284 |  |  |  |  |  |  |  |  |  |  |  |  |
| 6.MPAI6 | 0.362 | 0.411 | 0.420 | 0.366 | 0.569 |  |  |  |  |  |  |  |  |  |  |  |
| 7.MPAI7 | 0.369 | 0.352 | 0.363 | 0.335 | 0.452 | 0.517 |  |  |  |  |  |  |  |  |  |  |
| 8.MPAI8 | 0.162 | 0.166 | 0.237 | 0.243 | 0.219 | 0.267 | 0.317 |  |  |  |  |  |  |  |  |  |
| 9.MPAI9 | 0.242 | 0.298 | 0.306 | 0.155 | 0.385 | 0.369 | 0.404 | 0.489 |  |  |  |  |  |  |  |  |
| 10.MPAI10 | 0.286 | 0.343 | 0.328 | 0.144 | 0.338 | 0.378 | 0.409 | 0.450 | 0.534 |  |  |  |  |  |  |  |
| 11.MPAI11 | 0.323 | 0.383 | 0.362 | 0.291 | 0.386 | 0.402 | 0.457 | 0.409 | 0.559 | 0.681 |  |  |  |  |  |  |
| 12.MPAI12 | 0.226 | 0.251 | 0.162 | 0.093 | 0.170 | 0.197 | 0.194 | 0.287 | 0.326 | 0.323 | 0.377 |  |  |  |  |  |
| 13.MPAI13 | 0.214 | 0.232 | 0.212 | 0.224 | 0.183 | 0.262 | 0.248 | 0.326 | 0.286 | 0.366 | 0.415 | 0.609 |  |  |  |  |
| 14.MPAI14 | 0.234 | 0.291 | 0.166 | 0.275 | 0.214 | 0.256 | 0.277 | 0.296 | 0.243 | 0.314 | 0.400 | 0.480 | 0.783 |  |  |  |
| 15.MPAI15 | 0.264 | 0.294 | 0.306 | -0.028 | 0.335 | 0.372 | 0.333 | 0.225 | 0.371 | 0.442 | 0.395 | 0.313 | 0.391 | 0.388 |  |  |
| 16.MPAI16 | 0.310 | 0.328 | 0.313 | 0.102 | 0.406 | 0.460 | 0.392 | 0.228 | 0.342 | 0.413 | 0.381 | 0.245 | 0.218 | 0.224 | 0.488 |  |
| 17.MPAI17 | 0.245 | 0.287 | 0.311 | -0.107 | 0.420 | 0.408 | 0.312 | 0.191 | 0.360 | 0.416 | 0.365 | 0.218 | 0.160 | 0.131 | 0.462 | 0.617 |

**Table 2. Estimated unstandardized factor loadings of the configural, metric, scalar and residual invariant models.**

|  | Configural (S.E) | |  | Metric (S.E.) | |  | Scalar (S.E.) | |  | Residual (S.E.) | |
| --- | --- | --- | --- | --- | --- | --- | --- | --- | --- | --- | --- |
|  | Male | Female |  | Male | Female |  | Male | Female |  | Male | Female |
| Inability to Control Craving | | | | | | | | | | | |
| 1.MPAI1 | 1.000 (0.000) | 1.000 (0.000) |  | 1.000 (0.000) | 1.000 (0.000) |  | 1.000 (0.000) | 1.000 (0.000) |  | 1.000 (0.000) | 1.000 (0.000) |
| 2.MPAI2 | 1.256 (0.089) | 1.039 (0.098) |  | 1.239 (0.079) | 1.239 (0.079) |  | 1.216 (0.072) | 1.216 (0.072) |  | 1.155 (0.066) | 1.155 (0.066) |
| 3.MPAI3 | 1.339 (0.145) | 0.991 (0.124) |  | 1.328 (0.138) | 1.328 (0.138) |  | 1.242 (0.110) | 1.242 (0.110) |  | 1.177 (0.096) | 1.177 (0.096) |
| 4.MPAI4 | 0.660 (0.093) | 0.736 (0.098) |  | 0.640 (0.088) | 0.640 (0.088) |  | 0.746 (0.080) | 0.746 (0.080) |  | 0.697 (0.067) | 0.697 (0.067) |
| 5.MPAI5 | 1.541 (0.153) | 1.500 (0.160) |  | 1.526 (0.137) | 1.526 (0.137) |  | 1.538 (0.126) | 1.538 (0.126) |  | 1.519 (0.110) | 1.519 (0.110) |
| 6.MPAI6 | 1.861 (0.192) | 1.799 (0.202) |  | 1.821 (0.170) | 1.821 (0.170) |  | 1.897 (0.163) | 1.897 (0.163) |  | 1.826 (0.139) | 1.826 (0.139) |
| 7.MPAI7 | 1.496 (0.157) | 1.557 (0.160) |  | 1.489 (0.145) | 1.489 (0.145) |  | 1.526 (0.131) | 1.526 (0.131) |  | 1.519 (0.113) | 1.519 (0.113) |
| Anxiety and Feeling Lost | | | | | | | | | | | |
| 8.MPAI8 | 1.000 (0.000) | 1.000 (0.000) |  | 1.000 (0.000) | 1.000 (0.000) |  | 1.000 (0.000) | 1.000 (0.000) |  | 1.000 (0.000) | 1.000 (0.000) |
| 9.MPAI9 | 1.390 (0.146) | 1.566 (0.185) |  | 1.387 (0.146) | 1.387 (0.146) |  | 1.359 (0.120) | 1.359 (0.120) |  | 1.479 (0.116) | 1.479 (0.116) |
| 10.MPAI10 | 1.696 (0.198) | 2.189 (0.257) |  | 1.690 (0.197) | 1.690 (0.197) |  | 1.669 (0.163) | 1.669 (0.163) |  | 1.927 (0.161) | 1.927 (0.161) |
| 11.MPAI11 | 2.006 (0.233) | 2.447 (0.308) |  | 2.003 (0.233) | 2.003 (0.233) |  | 2.074 (0.210) | 2.074 (0.210) |  | 2.212 (0.189) | 2.212 (0.189) |
| Withdrawal and Escape | | | | | | | | | | | |
| 12.MPAI12 | 1.000 (0.000) | 1.000 (0.000) |  | 1.000 (0.000) | 1.000 (0.000) |  | 1.000 (0.000) | 1.000 (0.000) |  | 1.000 (0.000) | 1.000 (0.000) |
| 13.MPAI13 | 2.224 (0.263) | 2.488 (0.393) |  | 2.302 (0.265) | 2.302 (0.265) |  | 2.289 (0.239) | 2.289 (0.239) |  | 2.310 (0.216) | 2.310 (0.216) |
| 14.MPAI14 | 1.910 (0.203) | 1.508 (0.146) |  | 1.878 (0.184) | 1.878 (0.184) |  | 1.912 (0.177) | 1.912 (0.177) |  | 1.710 (0.124) | 1.710 (0.124) |
| Productivity Loss | | | | | | | | | | | |
| 15.MPAI15 | 1.000 (0.000) | 1.000 (0.000) |  | 1.000 (0.000) | 1.000 (0.000) |  | 1.000 (0.000) | 1.000 (0.000) |  | 1.000 (0.000) | 1.000 (0.000) |
| 16.MPAI16 | 1.227 (0.121) | 1.109 (0.140) |  | 1.199 (0.111) | 1.199 (0.111) |  | 1.183 (0.101) | 1.183 (0.101) |  | 1.169 (0.091) | 1.169 (0.091) |
| 17.MPAI17 | 0.999 (0.106) | 0.914 (0.106) |  | 0.983 (0.101) | 0.983 (0.101) |  | 0.981 (0.091) | 0.981 (0.091) |  | 0.960 (0.075) | 0.960 (0.075) |
